# Reduced Protein Expression of the Na^+^/Ca^2+^+K^+^-Exchanger (SLC24A4) in Apical Plasma Membranes of Maturation Ameloblasts of Fluorotic Mice

**DOI:** 10.1007/s00223-016-0197-4

**Published:** 2016-10-17

**Authors:** A. L. J. J. Bronckers, R. Jalali, J. Lytton

**Affiliations:** 1 Department of Oral Cell Biology, Academic Centre for Dentistry Amsterdam (ACTA), MOVE Research Institute, University of Amsterdam and VU University Amsterdam, Gustav Mahlerlaan 3004, 1081LA Amsterdam, The Netherlands; 2 Department of Biochemistry and Molecular Biology, Hotchkiss Brain Institute and Libin Cardiovascular Institute of Alberta, Cumming School of Medicine, University of Calgary, Calgary, AB T2N 4Z6 Canada

**Keywords:** Enamel fluorosis, Mechanism, Null mutation, Transport, Calcium

## Abstract

Exposure of forming enamel to fluoride results into formation of hypomineralized enamel. We tested whether enamel hypomineralization was caused by lower expression of the NCKX4/SLC24A4 Ca^2+^-transporter by ameloblasts. Three commercial antibodies against NCKX4 were tested on enamel organs of wild-type and *Nckx4*-null mice, one of which (a mouse monoclonal) was specific. This antibody gave a prominent staining of the apical plasma membranes of maturation ameloblasts, starting at early maturation. The layer of immuno-positive ameloblasts contained narrow gaps without immunostaining or with reduced staining. In fluorotic mouse incisors, the quantity of NCKX4 protein in ameloblasts as assessed by western blotting was not different from that in non-fluorotic ameloblasts. However, immunostaining of the apical plasma membranes of fluorotic ameloblasts was strongly reduced or absent suggesting that trafficking of NCKX4 to the apical membrane was strongly reduced. Exposure to fluoride may reduce NCKX4-mediated transport of Ca^2+^ by maturation stage ameloblasts which delays ameloblast modulation and reduces enamel mineralization.

## Introduction

Enamel fluorosis is a structural defect in enamel caused by too high intake of fluoride during development of the dentition. Fluorotic enamel is hypomineralized, and the surface contains white opacities that after eruption can develop into surface pits and grooves. In particular, the maturation stage of amelogenesis is sensitive to fluoride, and mineralization is incomplete in fluorotic enamel. How this occurs is still not clear.

Formation of the enamel takes place in two major stages, secretory and maturation stage [1]. During the maturation stage of amelogenesis, two types of functional ameloblasts can be recognized based on the structure of their apical membrane facing enamel: One type consists of ameloblasts with apical ruffle-ended membranes (RE) that secrete Ca^2+^ into slightly acidic (pH 6.2) enamel, and the other type has smooth-ended (SE) membranes that do not secrete Ca^2+^ and face enamel that is pH neutral. Each maturation ameloblast cyclically changes from RE mode into SE mode in a wave like fashion from apical to incisal end multiple times called modulation or pH cycling [1]. During modulation, enamel becomes increasingly mineralized. Exposure to fluoride delays modulation and extends the time that ameloblasts are in RE mode [2, 3].

Maturation ameloblasts secrete bicarbonates into the forming enamel to buffer protons that are released when apatite crystals are forming. Ameloblasts secrete bicarbonate in exchange for Cl^−^ present in enamel fluid [4]. In fluorotic mice, the Cl^−^ content in enamel is reduced suggesting that also the capacity of fluorotic ameloblasts to secrete bicarbonate is reduced [5–7]. Reduced secretion of buffer by ameloblasts could explain why the acidic RE bands in fluorotic enamel are wider [3, 4]. Ultrastructural detection of pyroantimonate precipitable Ca^2+^ in plasma membranes of ameloblasts suggested that secretory and RE ameloblasts but not SE ameloblasts transport Ca^2+^ across their membranes [8]. Exposure to F reduces the number of pyroantimonate precipitable Ca^2+^ in the intercellular space of secretory ameloblasts [9] suggesting that fluoride reduced Ca^2+^ transport is one factor responsible for hypomineralization of fluorotic enamel.

The K^+^-dependent Na^+^/Ca^2+^K^+^ Exchanger-4 (NCKX4/SLC24A4) is expressed in a number of tissues including brain, olfactorial cells [10–12] and ameloblasts where it was shown to be essential for full completion of enamel mineralization [13, 14]. The mouse NCKX4 is a 622-amino acid long protein with eleven predicted transmembrane helices that transports Ca^2+^ across the plasma membrane [9, 10]. Immunohistochemical studies showed that ameloblasts start expressing NCKX4 protein at mid-maturation [5, 15, 16].

The purpose of the present study was to examine whether hypomineralization of fluorotic enamel is associated with reduced protein expression of NCKX4 in ameloblasts.

## Materials and Methods

### Animals

Three-week-old C57Bl/J6 mice were purchased from Harlan (France), half of the group (*n* = 4) exposed to 100 ppm fluoride in drinking water for 6 weeks, the other group not as reported [4]. *Nckx4*-null mice (C57Bl/J6 background) and age-matched wild-type controls were raised as reported [10]. For western blotting, the mandibles of the mice including developing incisors were excised, slam-frozen and freeze-dried. Labial bone overlaying the incisors was removed to expose the enamel organ soft tissue which was carefully removed by microsurgery under a 4 × magnification and used for western blotting. For immunohistochemistry, lower jaws of *Nkcx4*-null and wild-type controls were excised and fixed by immersion in 10 % buffered formalin overnight, rinsed in PBS with 0.2 % formalin and shipped to Amsterdam for processing. Jaws were decalcified in 5 % EDTA pH 7.0 for 4–6 weeks, rinsed, dehydrated, embedded in paraffin and 7 µm-thick sagittal sections cut and mounted on glass slides.

### Antibodies

Three antibodies were purchased with the following specifications by the manufacturers: (1) Affinity purified polyclonal rabbit anti-NCKX4 from Protein Tech group Inc., (Chicago, Il, USA) (#18992-1-AP). It was raised against the N-terminal peptide sequence of human NCKX4 (NM_153646) (DTWRNRKLMAPVNGTQTAKNC; amino acids 57–77); (2) Affinity purified rabbit polyclonal anti-NCKX4 (Abcam, Cambridge, UK; #136968), raised against the peptide sequence GVSSKPLQNGRHENIENGNVPVENPEDPQQ (amino acids 360–389 of the human sequence Q8NFF2). (3) Mouse monoclonal IgG2b isotype (NeuroMab, UC Davis/NIH NeuroMab Facility, catalogue # N414/25), against a fusion protein of amino acids 246–424 of the human sequence (Q8NFF2), coding for the third intracellular loop of human NCKX4 isoform 3. According to the supplier this monoclonal antibody was validated on brains of *Nckx4*-null mutant mice and did not cross-react with NCKX2 or NCKX3. To test specificity of all three antibody species, we used *Nckx4*-null mutant mice from which exons 6 (amino acids 160–194) and 7 (amino acids 195–219) were excised which abolished protein expression of the entire 622 amino acid long NCKX4 protein [10]. Mouse anti-β-actin antibody was from Sigma-Aldrich, St. Louis, MO, USA.

### Western Blotting

Enamel organ extracts from *Nckx4*-null and matched wild-type control mice were probed by immunoblot. Freeze-dried enamel organs were dissolved under nonreducing condition in SDS loading buffer (NucleoSpin TriPrep; Macherey-Nagel, Bioke, Leiden, The Netherlands), and protein was measured with the BCA protein assay (Bio-Rad, Hercules, CA, USA). 5–10 μg of protein was loaded on SDS-PAGE in a 3–8 % *BIS*–*TRIS* NuPAGE gel (Thermo Fisher Scientific,Grand Island, NY, USA) with MOPSe as running buffer for 35 min at 200 V and electroblotted by an iBlot device (Invitrogen) on nitrocellulose membrane according to the manufacturer’s instructions. Blots were incubated with rabbit primary antibodies to NCKX4 (1:500) or mouse primary antibody to NCKX4 and mouse-β-actin antibody (1:1000; Sigma-Aldrich, St. Louis, MO, USA) overnight. IRDye 800CW-conjugated goat anti-rabbit IgG (H + L) highly cross-adsorbed (926–32211; LI-COR Biosciences, Lincoln, NE, USA), and IRDye 680CW-conjugated goat anti-mouse IgG (H + L) highly cross-adsorbed (926–32220; LI-COR) were applied as a second antibody for 90 min at room temperature (1:5000; LI-COR) prior to washing with PBS. Visualization and quantification were carried out with the OdysseyH scanner and software (LI-COR). Red color (for mouse anti-actin or mouse anti-NCKX4) was detected at a 680-nm wavelength, and a green color (for rabbit anti-NCKX4) at a 800-nm wavelength. For quantification, Odyssey software was used. Intensity values of the bands were normalized for actin and expressed as percentage of wild-type (100 %).

### Immunohistochemistry

Formalin-fixed paraffin sections were dewaxed, rehydrated, washed in phosphate buffered saline. After antigen retrieval in EDTA (10 mM) pH 9.0 for 3–6 h at 60 °C and blocking with blocking solution (Envision kit, Dakopatt Glostrup, Denmark) sections were incubated with primary antibodies (1:500) at 4 °C overnight, rinsed and incubated with anti-mouse IgG peroxidase conjugates or goat anti-rabbit IgG peroxidase conjugates (Envision kit). After washing staining was visualized by DAB solution (Envision kit) and counterstained with hematoxylin. All experiments were approved by the Committee for Animal Care (Vrije Universiteit Amsterdam; ACTA-12-01) and were carried out in accordance with the approved guidelines.

## Results

### Antibody Validation

**Fig. 1:**
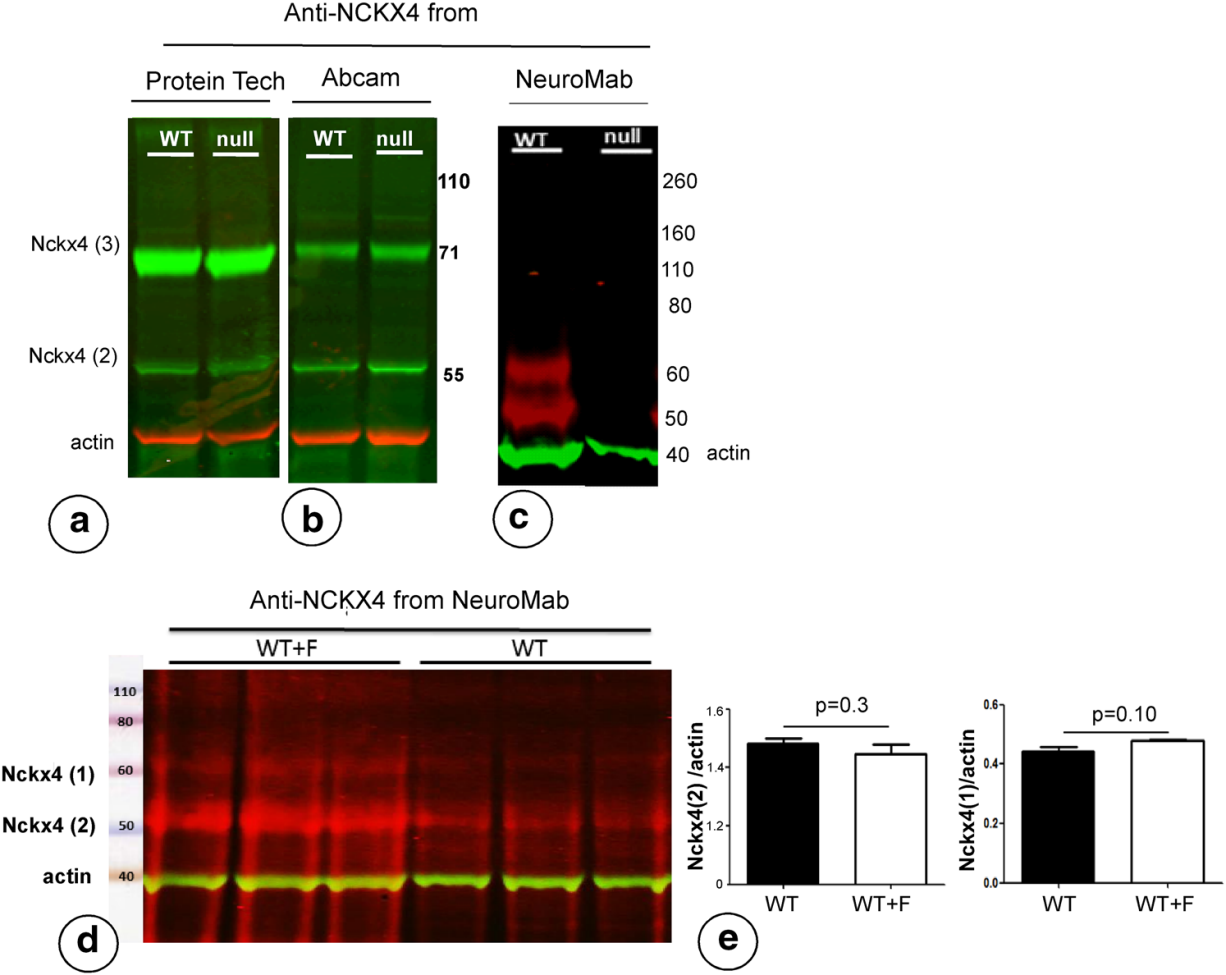
Western blots of enamel organ from *Nckx4*-null mice immunostained with three different antibodies to NCKX4. Tissues were stained with antibodies to NCKX4 from **a** Protein Tech (NCKX4 in green, ß-actin in red). **b** Abcam (NCKX4 in green, ß-actin in red) and **c** NeuroMab (NCKX4 in red, ß-actin in *green*). **d** Western blots of fluorotic and non-fluorotic wild-type ameloblasts stained with anti-NCKX4 (NeuroMab, NCKX4 in red; ß-actin in *green*) and **e** quantified after normalization to ß-actin. The 50 kD band contained almost fourfold more protein than the 60 kD band (Color figure online)

Blots of protein extracts from wild-type ameloblasts immunostained with antibodies from all three different suppliers showed a positive band around 50–55 kD (Fig. 1a–c). The polyclonal antibodies from Protein Tech (Fig. 1a) and Abcam (Fig. 1b) gave an additional positive band between 70–80 kD. The mouse monoclonal antibody from NeuroMab gave an additional band at ~60 kD (Fig. 1c).

When enamel organ extracts of *Nckx4*-null mice were immunoblotted, both polyclonal rabbit antibodies showed the same bands as in wild-type controls (Fig. 1a, b). The mouse monoclonal anti-NCKX4, however, failed to stain with the 50 and 60 kD bands in *Nckx4*-null enamel organs (Fig. 1c).

**Fig. 2:**
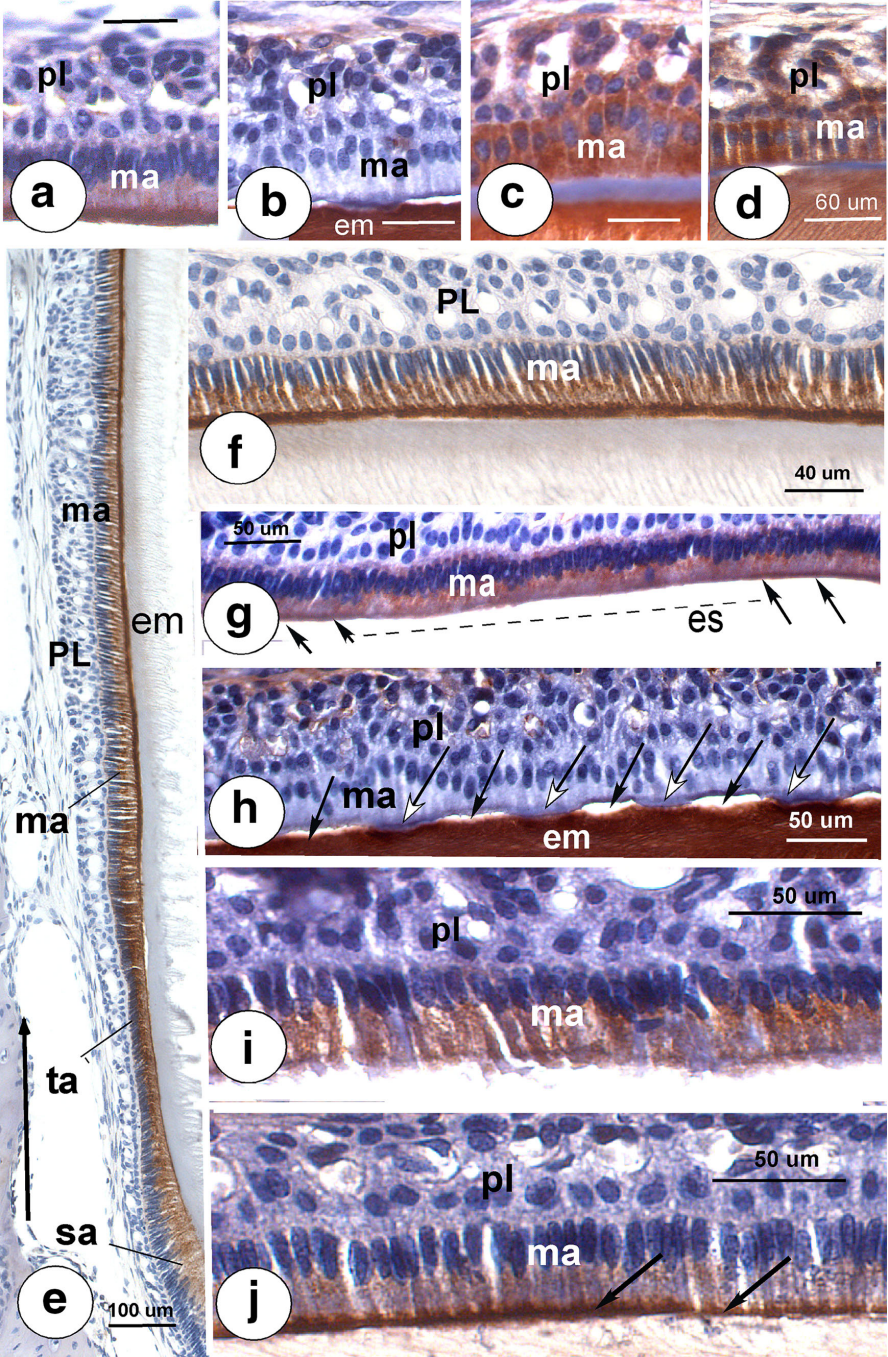
Validation of the antibodies to NCKX4 (**a**–**d**), developmental expression in wild-type incisors (**a**, **e**, **f**, **g** for NeuroMab antibody) and expression in fluorotic wild-type enamel organ (**i**, **j** NeuroMab antibody). **a** Apical membranes of wild-type maturation ameloblasts (ma) but not membranes of the papillary layer (pl) stain positive with mouse anti-NCKX4 (NeuroMab). **b**–**d** *Nckx4*-null maturation ameloblasts stained with anti-NCKX4 from NeuroMab (**b**), Protein Tec (**c**) and Abcam (**d**). All three antibodies stained to various degrees the extracellular enamel matrix when matrix was retained in the enamel space in *Nckx4*-null teeth. Similar variable stainings of forming enamel are also seen with other non-related antibodies in undecalcified (or partly decalcified) enamel sections or when primary antibodies are replaced by non-immuno-IgG, rabbit or mouse normal serum. This staining was considered *non*-*specific.* **e** Developmental expression of NCKX4 protein starts at late secretory stage ameloblasts (sa), continues in transitional ameloblasts (ta) and soon locates prominent in apical membranes of all early maturation ameloblasts (ma). *Large arrow* at the bottom points incisally.Pl papillary layer. **f** Detail of mid-maturation ameloblasts with strongly stained apical membranes. **g** Small group of cells underlined by a dotted line (likely SE ameloblasts) without pronounced apical staining in contrast to strong apical staining in neighbor cells (*arrows*). Es enamel space. **h** Shows negative staining in *Nckx4*-null ameloblasts stained with NeuroMab antibody, also showing periodic changes in attachment of the apical membrane to the enamel matrix (em). The *black arrows* point at local detachment of the apical membrane from the enamel; the *arrows* with *white arrow* heads point at plaque-like focal adhesions of the apical membrane to the enamel. Note these plaques stain *blue with hematoxylin*. **i** Shows fluorotic wild-type mouse ameloblasts without apical staining; **j** shows strong apical staining in non-fluorotic wild-type control ameloblasts

In tissue sections of wild-type ameloblasts, all three antibodies immunostained with enamel organ cells, the polyclonal antibodies as reported before [5]. The mouse monoclonals from NeuroMab gave a strong apical staining of maturation ameloblasts but did not stain papillary layer cells (Fig. 2a, e, f).

Tissue sections of *Nckx4*-null incisors did *not* react with antibody from NeuroMab (Fig. 2b, h) but stained with antibodies from Protein Tech (Fig. 2c) and Abcam (Fig. 2d).

Histology indicated that in *Nckx4*-null mice the developing enamel in incisors and erupted molars retained substantial amounts of matrix proteins not found after mid- maturation in the wild-type incisors (not shown). Maturation ameloblasts and adjacent papillary layer were less well organized (Fig. 2h) than in wild-type controls (Fig. 2a, f). In *Nckx4*-null incisors groups of maturation ameloblasts were seen to locally detach from the enamel surface, while neighbor groups remained attached to the enamel by plaque-like adhesive, regularly spaced structures that stained blue with hematoxylin (Fig. 2h).

The papillary layer of wild-type enamel organs weakly immunostained with the Abcam and Prot Tech antibodies (see reference 5) but moderately to more strongly in papillary layer cells of *Nckx4*-null mice (Fig. 2c, d).

#### Developmental Expression of NCKX4 Protein in Incisors of Wild-Type Mice

NeuroMab mouse monoclonal anti-NCKX4 gave the first (weak) intracellular staining in late secretory–transitional stage ameloblasts (Fig. 2e), and soon heavy staining was seen over the apical membranes of early maturation stage ameloblasts. Small groups of maturation ameloblasts with negative- or weakly-stained cells without pronounced apical staining were also observed presumably representing a group of SE ameloblasts (Fig. 2g). The papillary layer (Fig. 2a, e, f), and odontoblasts (not shown) did not stain with the mouse anti-NCKX4.

#### Fluorotic Ameloblasts Show Far Less Protein Staining in Apical Membranes

Staining of the apical membranes of fluorotic wild-type ameloblasts with mouse anti-NCKX4 was consistently weak or absent (Fig. 2i) with strong intracellular staining. Non-fluorotic controls revealed strong apical staining (Fig. 2j).

Densitometric measurements of both 50 and 60 kD bands positive for NCKX4 (Fig. 1d) on the western blots normalized for ß-actin showed that fluorotic ameloblasts contained the same amount of immunoreactive protein as non-fluorotic ameloblasts (Fig. 1e).

## Discussion

Previous studies localized NCKX4 in enamel organs with antibodies that were not validated for specificity [5, 14–16]. In the course of the present study, *Nckx4*-null tissues became available which enabled us to validate the antibodies previously used. Our data show that only the NeuroMab mouse monoclonal antibody reacted specifically with NCKX4, shown by the failure of enamel organs of *Nckx4*-null mice to stain with anti-NCKX4 when tested both on western blots and in tissue sections. The two polyclonal antibodies stained positive on *Nckx4*-null enamel organs indicating these antibodies also react with other epitopes than NCKX4. The overall staining patterns of both polyclonal antibodies on tissue sections of wild-type mice ameloblasts were, however, not much different from that of the monoclonal antibodies except that the monoclonal did not stain the papillary layer or odontoblasts and was expressed earlier.

Enamel organs of rodents express all six isoforms of the NCKX/SLC24A4 family from which NCKX4 is expressed the highest [15]. A search in NCBI gen bank indicated that several of these isoforms are potentially expressed in the same range as NCKX4 (500–700 amino acid long proteins). The Abcam and Protein Tech anti-NCKX4 may recognize some of these isoforms, especially when these transporters are attempting to compensate for the loss of NKCX4. This may result in their accumulation in *Ncxk4* null mice explaining elevated staining of ameloblasts and papillary layer with both polyclonal anti-NCKX4. The present data illustrate again the importance of validating the specificity of antibodies on null mutant tissues whenever possible.

Various Ca^2+^ transporters and exchangers have been identified in ameloblasts that could play a role in secretion of Ca^2+^ into the enamel space to form apatites. These include Plasma Membrane Ca^2+^ ATP-ases (PMCA) [17–19], the Na^+^/Ca^2+^ exchangers NCX1 and NCX3 [20, 21] and NCKX4 [13–15]. Compared to the plasma membrane ATPases and NCX1/ NCX3 that are expressed in secretory stage and continued expression at the same (NCX1) or reduced (NCX3) level at maturation stage [21], the expression of NCKX4 starts at late secretion and rapidly increases at maturation stage. NCKX4 also has a transport capacity much higher than PMCA and NCX’s [20]. Null mutation of *Nckx4* severely reduces enamel mineralization at maturation stage [13]. Collectively, we conclude from the present and published data that NCKX4 is a key Ca^2^ exchanger responsible for *mineral deposition* during maturation stage of forming enamel.

The typical periodic detachment of maturation ameloblasts from the enamel surface in *Nckx4*-null mice at some locations alternating with small groups of cells that remain attached to the enamel resembles the effect of *Sppl2A* null mutation on enamel, an intramembrane protease residing in lysosomes and late endosomes that cleaves type II-oriented transmembrane proteins [22] Local detachment of maturation ameloblasts from the enamel surface may reduce endocytosis which could explain matrix retention in *Nckx4*-null enamel.

The majority (80%) of maturation stage ameloblast in rat incisors at a given time are RE ameloblasts, and the remaining 20% are SE ameloblasts [1]. Radioautographic studies with ^45^Ca^2+^ [23] and ultrastructural detection of Ca^2+^ antimonate precipitates in plasma membranes [8] presented evidence that RE ameloblasts but not SE ameloblasts actively transport Ca^2+^ into the enamel space. In the ameloblast layer of non-fluorotic wild-type mice, narrow gaps of immunonegative staining for NCKX4 were noted similar as for AE2 [24], resembling groups of SE ameloblasts. Reduced or no immunostaining of SE ameloblasts or in small groups of ameloblasts that are assumed to be SE cells has been reported for a variety of proteins and including NCKX4 [5], Ae2 [25]; calbindin 9 k and 28 k [26], cyclin M4/Cnnm4 [27], transferrin-R [28], IGF1, IGF2, IGF1-R and IGF-R2 [29], HSP-25 [30] and v-H-ATPase [31]. Takano and Ozawa [23] concluded that “SE ameloblasts are formed from RE ameloblasts wich have become inactive metabolically and here the exhausted cytoplasmic organelles seem to be renewed and reactivated”. This concept is well in line with the reduced expression of proteins in the negative gaps. Consequently, very likely the negative immunostained gaps represent SE cells.

The quantity of Nckx4 protein in *fluorotic* mouse enamel found by western blots was not different from that of non-fluorotic enamel. Immunohistochemistry showed that the apical membranes of fluorotic maturation stage ameloblasts stained not or far less for NCKX4, than those in non-fluorotic controls. Fluorotic enamel is severely hypomineralized [4, 6]. The present data suggest that in fluorotic maturation stage ameloblasts the transport and incorporation of NCKX4 into the apical membrane is impaired which will likely reduce influx of Ca^2+^ into enamel.

In fluorotic teeth also modulation is changed. The transformation of slightly acidic bands in enamel (below RE ameloblasts) into neutral bands (below SE ameloblasts) is delayed [2, 3].

We have proposed that RE ameloblasts will transform into SE ameloblasts by gradual acidification of the enamel or by physico-chemical changes associated with acidification [5]. Above a critical value, these changes trigger the transformation of the Ca^2+^ transporting RE ameloblasts into non-Ca^2+^ secreting SE ameloblasts. With the present results, we explain the fluorotic effect on modulation by *reduction of Ca* ^*2*+^ *transport* due to the decrease in the number of NCKX4 molecules incorporated into the apical membrane of fluorotic ameloblasts. Conceivably this will reduce Ca^2+^ transport into fluorotic enamel, lower mineral formation which in turn reduces acid production. Less acidification results in a longer time to reach the critical value to trigger the transition of RE ameloblasts into SE ameloblasts. This delay widens the acid RE bands in enamel and results in a diffuse hypomineralization of fluorotic enamel.
